# A generalization of Ekeland’s variational principle by using the *τ*-distance with its applications

**DOI:** 10.1186/s13660-017-1435-7

**Published:** 2017-07-31

**Authors:** AP Farajzadeh, S Plubtieng, A Hoseinpour

**Affiliations:** 1 Department of Mathematics, Razi University, Kermanshah, 67149 Iran; 2 Department of Mathematics, Faculty of Science, Naresuan University, Phitsanulok, 65000 Thailand

**Keywords:** *τ*-distance, Ekeland’s variational principle, equilibrium problem, bounded below, lower semicontinuous function

## Abstract

In this paper, a new version of Ekeland’s variational principle by using the concept of *τ*-distance is proved and, by applying it, an approximate minimization theorem is stated. Moreover, by using it, two versions of existence results of a solution for the equilibrium problem in the setting of complete metric spaces are investigated. Finally some examples in order to illustrate the results of this note are given.

## Introduction

Ekeland’s variational principle was first expressed by Ekeland [1, 2] and developed by many authors and researchers. Tataru in [3] defined the concept of Tataru’s distance and using it proved the generalization of Ekeland’s variational principle. Afterwards in 1996, Kada *et al.* in [4] stated the concept of w-distance and extended Ekeland’s variational principle. The concept of *τ*-distance which is a generalization of w-distance and Tataru’s distance was first introduced by Suzuki. He also improved the concept of Ekeland’s variational principle; see [5]. Over the last few years, several authors have studied Ekeland’s variational principle for equilibrium problems under different conditions; see, for instance, [6, 7]. In these papers, the authors studied the equilibrium version of Ekeland’s variational principle to get some existence results for equilibrium problems in both compact and noncompact domains. In 2007, Bianchi *et al.* [8] introduced a vector version of Ekeland’s principle for equilibrium problems. They studied bifunctions defined on complete metric spaces with values in locally convex spaces ordered by closed convex cones and obtained some existence results for vector equilibria in compact and noncompact domains. However, several authors have made efforts to get new existence results in the studies of equilibrium problems, for instance, [9–11].

The purpose of this paper is to study equilibrium problem to get some existence results. In fact, we first recall the concept of *τ*-distance on a complete metric space and then by using it a new version of Ekeland’s variational principle by using the concept of *τ*-distance is proved and, by applying it, an approximate minimization theorem is stated. Moreover, as an application, two versions of existence results of a solution for the equilibrium problem in the setting of complete metric spaces are investigated. Finally some examples in order to illustrate the results of this note are given.

## Ekeland’s variational principle

In this section, we will rely on a new version of Ekeland’s variational principle which improves the corresponding result, replacing a meter by *τ*-distance [5], was obtained by the authors of [6]. We need the following preliminaries in the sequel.

### Definition 2.1

[5]

Let $(X,d)$ be a metric space. Then a function $p:X\times X\rightarrow \mathbb{R}$ is called *τ*-distance on *X* if

$\tau_{1}$:
$p(x,z)\leq p(x,y)+p(y,z)$, for all $x,y,z\in X$,

moreover, there exists a function $\eta:X\times \mathbb{R}^{+}\rightarrow \mathbb{R}^{+}$ which is concave and continuous in its second variable and satisfying the following conditions:

$\tau _{2}$:
$\eta (x,0)=0$ and $\eta (x,t)\geq t$, for all $x\in X$, $t\in \mathbb{R}^{+}$,
$\tau _{3}$:
$\lim_{n} x_{n}=x$ and $\lim_{n} \sup \{\eta (z _{n},p(z_{n},x_{m}))\mid m\geq n\}=0$ imply $p(w,x)\leq \liminf_{n} p(w,x _{n})$, for all $w\in X$,
$\tau _{4}$:
$\lim_{n} \sup \{p(x_{n},y_{m})\mid m\geq n\}=0$ and $\lim_{n}\eta (x_{n},t_{n})=0$ imply $\lim_{n}\eta (y_{n},t_{n})=0$,
$\tau _{5}$:
$\lim_{n}\eta (z_{n},p(z_{n},x_{n}))=0$ and $\lim_{n}\eta (z_{n},p(z_{n},y_{n}))=0$ imply $\lim_{n} d(x_{n},y_{n})=0$.

### Example 2.2

Let $X=[0,\infty ]$ and *d* be the Euclidean metric on *X*. Consider the function $p:X\times X\rightarrow \mathbb{R}$ defined by $p(x,y)=\vert y\vert $, for all $x,y\in X$. The function *p* is a *τ*-distance. Clearly, condition $\tau_{1}$ holds. To check the conditions $\tau_{2}-\tau _{5}$, we define a function $\eta:X\times \mathbb{R}^{+}\rightarrow \mathbb{R}^{+}$ by $\eta (x,t)=t$, for all $x\in X$ and $t\in \mathbb{R}^{+}$. Checking the conditions $\tau_{2}-\tau_{4}$ is straightforward. To prove $\tau_{5}$, let $\{x_{n}\}$, $\{y_{n}\}$ and $\{z_{n}\}$ be sequences in *X* such that $\lim_{n}\eta (z_{n},p(z _{n},x_{n}))=0$ and $\lim_{n}\eta (z_{n},p(z_{n},y_{n}))=0$. Hence, $\lim_{n}p(z_{n},x_{n})=0$ and $\lim_{n}p(z_{n},y_{n})=0$. Consequently,

$$\begin{aligned} d(x_{n},y_{n}) =&\vert x_{n}-y_{n} \vert \leq \vert x_{n}\vert +\vert y_{n}\vert \\ =& p(z_{n},x_{n})+p(z_{n},y_{n})=0. \end{aligned}$$

Thus, $\lim_{n}d(x_{n},y_{n})=0$. This completes the proof of $\tau_{5}$.

The next result plays a key role in proving the theorem concerned with Ekeland’s variational principle.

### Proposition 2.3

[5]

*Let* *X* *be a complete metric space*, *p* *be the* *τ*-*distance on* *X* *and* $f:X\rightarrow (-\infty,\infty ]$ *be proper*, *i*.*e*., $f\neq \infty $, *lower semicontinuous and bounded from below*. *Define* $Mx=\{y\in X\mid f(y)+p(x,y)\leq f(x)\}$, *for all* $x\in X$. *Then*, *for each* $u\in X$ *with* $Mu\neq \emptyset $, *there exists* $x_{0}\in Mu$ *such that* $Mx_{0}\subset \{x_{0}\}$. *In particular*, *there exists* $y_{0}\in X$ *such that* $My_{0}\subset \{y_{0}\}$.

Now we are ready to state a new version of Ekeland’s variational principle.

### Theorem 2.4

*Let* $(X,d)$ *be a complete metric space*, *p* *be the* *τ*-*distance on* *X*, *and* $f:X\times X\rightarrow (-\infty, +\infty ]$ *be a proper lower semicontinuous in its second variable and bounded from below function which* $f(t,t)=0$, *for all* $t\in X$. *Then*, *for all* $\varepsilon >0$ *and for all* $x_{0}\in X$ *with* $p(x_{0},x_{0})=0$, *there exists* $\overline{x}\in X$ *such that*

$$\begin{aligned} \textstyle\begin{cases} f(x_{0},\overline{x})+\varepsilon p(x_{0},\overline{x})\leq 0, \\ f(\overline{x},x)+\varepsilon p(\overline{x},x)>0, &\forall x\in X, x\neq \overline{x}. \end{cases}\displaystyle \end{aligned}$$

### Proof

Without loss of generality we can choose $\varepsilon =1$. Define the function $F:X\rightarrow 2^{X}$ by

$$F(x)=\bigl\{ y\in X\mid f(x,y)+p(x,y)\leq 0\bigr\} . $$

If $x_{0}\in X$ such that $p(x_{0},x_{0})=0$, then $x_{0}\in F(x_{0})$ and hence $F(x_{0})\neq \emptyset $. Thus, as a consequence of the Proposition 2.3, there exists $\overline{x}\in F(x_{0})$ such that $F(\overline{x})\subset \{\overline{x}\}$. Hence, it follows from $\overline{x}\in F(x_{0})$ that

$$f(x_{0},\overline{x})+p(x_{0},\overline{x})\leq 0. $$

Also the inclusion $F(\overline{x})\subset \{\overline{x}\}$ implies that

$$f(\overline{x},x)+p(\overline{x},x)>0,\quad \forall x\neq \overline{x}. $$

This completes the proof. □

The following example illustrates Theorem 2.4.

### Example 2.5

Let $X=[0,\infty ]$ and *d* be the Euclidean metric on *X*. Consider the function $p:X\times X\rightarrow \mathbb{R}$ defined by $p(x,y)=\vert y\vert $, for all $x,y\in X$. The function *p* is a *τ*-distance (see Example 2.2). Define the function $f:X\times X\rightarrow \mathbb{R}$ by $f(x,y)=\sin (y-x)$, for all $x,y\in X$. Obviously, *f* satisfies all the conditions of Theorem 2.4 and so if we take $x_{0}=0$ then $\overline{x}=0$ is a candidate which fulfils in the conclusion of Theorem 2.4. Similarly, in Theorem 2.4 for $x_{0}=\frac{ \pi }{2}$ we can obtain $\overline{x}=0$.

### Remark 2.6

Note that the authors of [6] could generalize Theorem 2.1 in [7] by replacing a norm by a metric. In the following, we also extend the main result of [6] (an extension of Theorem 2.1 in [7]) by substituting a meter by a *τ*-distance. Since each distance (meter) defines a *τ*-distance and Example 2.2 shows that the converse is not generally true, such a generalization is a real extension. Hence, Example 2.5 cannot be solved using the mentioned methods in [6, 7].

As a consequence of Theorem 2.4 we present the next result which is an extension of the corresponding results of [6] and [7], respectively, from metric space and normed space to the *τ*-distance.

### Theorem 2.7

*Let* $(X,d)$ *be a complete metric space*, *p* *be the* *τ*-*distance on* *X* *and* $g:X\longrightarrow (-\infty, +\infty ]$ *be proper*, *lower semicontinuous and bounded from below*. *Assume that*, *for any* $\varepsilon >0$, *there is* $x_{0}\in X$ *such that* $\inf_{x \in X}g(x)>g(x_{0})+\varepsilon $, $p(x_{0},x_{0})=0$ *and* $p(x_{0},x)>0$, *for all* $x\in X$ *with* $x\neq x_{0}$. *Then there exists* $\overline{x}\in X$ *such that*, *for all* $x\in X$ *with* $x\neq \overline{x}$, *we have*

1 $$\begin{aligned}& g(x)-g(\overline{x})>\varepsilon, \end{aligned}$$

2 $$\begin{aligned}& g(x)-g(\overline{x})+\varepsilon p(\overline{x},x)>0. \end{aligned}$$

### Proof

Let $\varepsilon >0$ be given. Define $f(x,y)=g(y)-g(x)$, for all $x,y\in X$. It is straightforward to check that *f* satisfies all the assumptions of Theorem 2.4 and hence there exists $\overline{x}\in X$ such that

3 $$\begin{aligned}& f(x_{0},\overline{x})+\varepsilon p(x_{0},\overline{x}) \leq 0, \end{aligned}$$

4 $$\begin{aligned}& f(\overline{x},x)+\varepsilon p(\overline{x},x)>0, \quad \forall x \in X,x\neq \overline{x}. \end{aligned}$$

From (4), we get

$$g(x)-g(\overline{x})+\varepsilon p(\overline{x},x)>0,\quad \forall x \in X,x\neq \overline{x}. $$

Thus, the inequality (2) holds. Let us now prove the inequality given by (1). On the contrary if

5 $$ f(\overline{x},x)=g(x)-g(\overline{x})< \varepsilon,\quad \mbox{for some } x\in X \mbox{ with } x\neq \overline{x}, $$

then it follows from (3) and the hypothesis of the theorem that

6 $$ g(\overline{x})-g(x_{0})+\varepsilon p(x_{0},\overline{x})\leq 0\quad \Rightarrow \quad g(x_{0})-g( \overline{x})\geq \varepsilon p(x_{0}, \overline{x})>0. $$

Also, it concludes from (5) that

$$\begin{aligned} \varepsilon >g(x)-g(\overline{x})>\inf_{x\in X}g(x)-g( \overline{x})>g(x _{0})-g(\overline{x})+\varepsilon. \end{aligned}$$

Hence, $g(x_{0})-g(\overline{x})<0$, which is contradicted by (6). This completes the proof. □

## Ekeland’s variational principle for equilibrium problems

Let *X* be a given set and $f:X\times X\rightarrow \mathbb{R}$ be a given function. An equilibrium problem is finding $\overline{x}\in X$ such that $f(\overline{x},y)\geq 0$, for all $y\in X$. We may abbreviate this problem with EP from now on. In this section, we intend to provide sufficient conditions to solve the EP using the new version of Ekeland’s variational principle and the concept of *τ*-distance.

The following result is a new version of Corollary 2.1 of [6] which guarantees the existence of a solution of EP in complete metric spaces with the notion of *τ* distance.

### Theorem 3.1

*Suppose that the assumptions of Theorem* 2.4 *are satisfied*. *Moreover*, *for every* $x\in X$ *with* $\inf_{y\in X}f(x,y)<0$, *there exists* $z\in X$ *such that* $z\neq x$ *and* $f(x,z)+ p(x,z)\leq 0$. *Then the EP has at least one solution*.

### Proof

By Theorem 2.4, there exists $\overline{x}\in X$ such that

7 $$\begin{aligned} f(\overline{x},y)+ p(\overline{x},y)>0, \quad \forall y\in X, y\neq \overline{x}. \end{aligned}$$

We claim that *x̅* is a solution of EP. Otherwise, there exists $y\in X$ such that $f(\overline{x},y)<0$. Hence, $\inf_{y \in X}f(\overline{x},y)<0$. According to the assumption of theorem, there exists $z\in X$ such that $z\neq \overline{x}$ and $f( \overline{x},z)+ p(\overline{x},z)\leq 0$, which is a contradiction to (7) and it completes the proof. □

In the following, using some results in [6, 7, 9, 12], we obtain two following theorems stating the existence of solutions of EP in two cases. In the first case, we discuss the existence of solutions of EP in a compact metric space and in the next one, we provide some conditions for the existence of solutions in a noncompact metric space.

### Theorem 3.2

*Beside the assumptions of Theorem* 2.4, *assume that* *X* *is a compact metric space and for each* $y\in X$ *the functions* $x\to f(x,y)$ *and* $x\to p(x,y)$ *are upper semicontinuous*. *Then the solution set of EP is nonempty and compact*.

### Proof

By Theorem 2.4, for all $n\in \mathbb{N}$, the positive integer numbers, there exists $x_{n}\in X$ such that

8 $$\begin{aligned} f(x_{n},y)+\frac{1}{n} p(x_{n},y)>0,\quad \forall y\in X, y\neq x_{n} . \end{aligned}$$

Since *X* is compact, the sequence $\{x_{n}\}$ has a convergent subsequence in *X*, say $\{x_{n_{k}}\}$. Hence, there exists $\overline{x}\in X$ such that $x_{n_{k}}\rightarrow \overline{x}$ as $k\rightarrow \infty $. It follows from (8) and upper semicontinuity of the functions *f* and *p* in the first variable that

$$\begin{aligned} f(\overline{x},y) \geq 0,\quad \forall y\in X. \end{aligned}$$

Hence, *x̅* is a solution of EP. The upper semicontinuity of *f* in the first variable guarantees the compactness of the solution set of EP. This completes the proof. □

Remark that the result of Theorem 3.2 is valid if we replace the upper semicontinuity of *p* in the first variable by the bounded above function $x\to p(x,y)$, for all $y\in X$.

In the next theorem, we present an existence result for the noncompact case.

### Theorem 3.3

*Assume that all assumptions of Theorem* 2.4 *hold*. *Let the function* *p* *be lower semicountinuous in the first variable*. *In addition*, *there is a compact set* $K\subseteq X$ *such that*

9 $$\begin{aligned} \forall x\in X\backslash K, \exists y\in K\quad \textit{with } p(y,x_{0})\leq p(x,x_{0}) \textit{ and } f(x,y)\leq 0, \end{aligned}$$

*where* $x_{0}$ *is an arbitrary element of* *X* *with* $p(x_{0},x_{0})=0$, *and*

10 $$\begin{aligned} f(x,y)\leq f(x,z)+f(z,y),\quad \forall x,y,z\in X. \end{aligned}$$

*Then EP has at least one solution*.

### Proof

Define $F:X\rightarrow 2^{X}$ by

11 $$\begin{aligned} F(x)= \bigl\{ y\in X\mid p(y,x_{0})\leq p(x,x_{0}) \text{ and } f(x,y) \leq 0 \bigr\} . \end{aligned}$$

Obviously, for all $x\in X$, $F(x)\neq \emptyset $, note $x\in F(x)$, and $F(x)$ is a closed set, notice that *p* and *f*, respectively, are lower semicontinuous in the first variable and the second variable. It is easy to see that, for any $x,y\in X$, if $y\in F(x)$, then $F(y)\subseteq F(x)$. Since *K* is a compact set, all assumptions of Theorem 3.2 hold for the function $f:K\times K\rightarrow \mathbb{R}$, and so there exists $x_{k}\in K$ such that

12 $$\begin{aligned} f(x_{k},y)\geq 0,\quad \forall y\in K. \end{aligned}$$

We claim that $x_{k}$ is a solution of EP on *X*. Assume that this assertion is not true, therefore

13 $$\begin{aligned} \exists\, \overline{x}\in X \quad \mbox{such that } f(x_{k}, \overline{x})< 0. \end{aligned}$$

We note that $F(\overline{x})\cap K$ is nonempty, because of we have two cases, the first case $\overline{x}\in K$ and so $\overline{x}\in F( \overline{x})\cap K$, and the second case $\overline{x}\notin K$. Then by (9) there exists $y\in K$ such that $p(y,x_{0})\leq p(x,x _{0})$ and $f(x,y)\leq 0$, and so $y\in F(\overline{x})\cap K$. This completes the proof of the assertion.

Set

14 $$\begin{aligned} a=\min_{y\in F(\overline{x})\cap K} p(y,x_{0}). \end{aligned}$$

Based on the assumption of theorem, the minimum is obtained, note $F(\overline{x})\cap K$ is a nonempty compact set and *p* is lower semicontinuous in the first variable. Thus, there exists $x_{1}\in F( \overline{x})\cap K$ such that $p(x_{1},x_{0})=a$. Hence $x_{1}\in F( \overline{x})$, and so it follows from the definition of $F( \overline{x})$ that $f(\overline{x},x_{1})\leq 0$ and, according to (13), we obtain

$$\begin{aligned} f(x_{k},x_{1})\leq f(x_{k},\overline{x})+f( \overline{x},x_{1})< 0, \end{aligned}$$

which is contradicted by $x_{1}\in K$, because of the relation given by (12). This completes the proof. □

## Conclusion

In the present paper, we study the vectorial form of Ekeland’s variational principle by using the concept of *τ*-distance. We obtain some existence results for the equilibrium problems in the setting of complete metric spaces in the cases of compact and noncompact spaces.
